# Characterization of the stanford integrated psychosocial assessment for transplant for heart, liver, and kidney transplant candidates in Japan

**DOI:** 10.1186/s13030-023-00281-6

**Published:** 2023-07-17

**Authors:** Kosuke Takano, Hidehiro Oshibuchi, Sayaka Kobayashi, Junko Tsutsui, Satoko Ito, Rumiko Kamba, Rie Akaho, Katsuji Nishimura

**Affiliations:** 1 Department of Psychiatry, Tokyo Women’s Medical University, 8-1, Kawada-cho, Shinjuku-ku, Tokyo, 162-8666 Japan; 2 Department of Psychiatry, Saitama Medical Center, Saitama Medical University, Kawagoe, Kamoda, Kawagoe-shi, Saitama, Saitama 1981, 350-8550 Japan; 3 Faculty of Human Science, Denen-chofu University, 3-4-1 Higashiyurigaoka, Asao-ku, Kawasaki City, Kawasaki-shi, Kanagawa 215-8542 Japan

**Keywords:** Organ-specific, Pre-transplant evaluation, Post-transplant outcomes, Psychosocial support, Stanford Integrated Psychosocial Assessment for transplantation, Transplant recipient

## Abstract

**Background:**

The Stanford Integrated Psychosocial Assessment for Transplantation (SIPAT) is a comprehensive psychosocial assessment proven useful for predicting the outcomes of organ transplantation that is expected to be useful in Japan. However, the characteristics of organ-specific SIPAT scores for organ transplant recipient candidates in Japan are unclear and, to date, the SIPAT has not been properly utilized in clinical practice. The purpose of this study was to present basic data that can be used to establish the relation between SIPAT scores and post-transplantation psychosocial outcomes as well as organ-specific outcomes.

**Methods:**

This study included 167 transplant recipient candidates (25 heart, 71 liver, and 71 kidney) who completed a semi-structured interview based on the Japanese version of SIPAT (SIPAT-J) prior to transplantation. The differences between organs in terms of SIPAT scores and differences in SIPAT scores based on demographic data were comparatively analyzed.

**Results:**

The total SIPAT scores were higher for liver recipient candidates than for heart recipient candidates (P = .019). Regarding the subscales, SIPAT B (social support system) scores were higher for liver and kidney recipient candidates than for heart recipient candidates (P = .021), whereas SIPAT C (psychological stability and psychopathology) scores were higher for liver recipient candidates than for kidney recipient candidates (P = .002). Recipient candidates with a history of psychiatric treatment and those who were unemployed had higher SIPAT scores, regardless of the transplant organ, than recipient candidates without a history of psychiatric treatment and those who were employed (P < .001, P = .016, respectively).

**Conclusions:**

There were notable differences in the total SIPAT-J and subscale scores among the liver, heart, and kidney recipient candidates. Each organ was associated with specific psychosocial issues that should be addressed before transplantation. Interventions such as information provision and patient education based on SIPAT assessment results for each organ may improve recipient post-transplant outcomes.

## Background

Although organ transplantation improves the prognosis of patients with end-stage organ failure, post-transplant physical and psychological outcomes are influenced by the recipient’s psychosocial factors prior to transplant surgery [1–5]. Therefore, psychosocial assessment of organ recipients is an integral part of the pre-transplant evaluation process, and the results can be expected to help predict post-transplant outcomes. Pre-transplant psychosocial evaluation to accurately assess the risk of post-transplant outcomes should be comprehensive, including the assessment of cognitive, behavioral, psychological, and social risk factors that may influence the transplant process and post-transplant outcomes [6].

One tool for the psychosocial assessment of recipients of both solid organ and hematopoietic cell transplants is the Psychosocial Assessment of Candidates for Transplant (PACT) [7, 8]. Low PACT scores have been shown to predict poor outcomes in patients who undergo hematopoietic cell, lung, and kidney transplantation [9–11] and also predict the occurrence of psychiatric disorders after liver transplantation in Japanese patients [12]. In 2017, the Japanese version of the PACT (J-PACT) was developed and tested for reliability and validity, although only allogeneic hematopoietic cell transplant candidates were evaluated in the process [13]. However, with only eight items, the PACT may be too simple to be a comprehensive assessment tool.

Maldonado et al. [14] developed the Stanford Integrated Psychosocial Assessment for Transplantation (SIPAT), a comprehensive psychosocial assessment tool applicable to all transplanted organs, and subsequent studies have demonstrated its utility in predicting outcomes for solid organ transplantation [15–20], hematopoietic stem cell transplantation [21, 22], and ventricular assist device implantation [23–25]. The SIPAT has been translated into Spanish, Italian, and Thai; all have demonstrated excellent inter-rater reliability and internal consistency [21, 26, 27]. We, therefore, translated the SIPAT into Japanese, validated its inter-rater reliability and internal consistency, and created a Japanese version of SIPAT (SIPAT-J) [6]. The SIPAT was designed to standardize the psychosocial assessment of transplant recipient candidates and to quantify the appropriateness of various transplants. It assesses a total of 18 psychosocial risk factors, with each item weighted according to the results of a review of previous studies; the total SIPAT score ranges from 0 to 110, with higher scores indicating a higher risk of negative psychosocial outcomes [6, 14]. Future widespread use of the SIPAT-J in Japan is anticipated.

Transplantation care in Japan is unique compared with that in other countries. In Japan, there are very few organ donations from patients after brain and cardiac death, and the waiting period for recipients is long [28]. Additionally, more than 90% of kidney and 80% of liver transplants are living donor transplants, and the percentage of living donor transplants among all organ transplants is much higher in Japan than in other countries [29]. Although the SIPAT has been translated into multiple languages and used in many countries [21, 26, 27], different circumstances surrounding transplantation medicine may lead to different psychosocial results among the recipient candidates in different countries. Therefore, the unique environment in Japan makes it difficult for the SIPAT to be properly utilized because there are currently no basic data for SIPAT-J scores to be applied to organ transplant recipient candidates. Additionally, the SIPAT is a psychosocial screening tool that can be used before transplantation and regardless of the transplant organ, such as the heart, liver, kidney, or lung. The SIPAT compares favorably with the PACT, but it is a more comprehensive rating scale and shows some significant advantages, including detailed descriptions regarding social support; substance abuse, use, and recidivism risk; knowledge regarding illness and the transplantation process; the effects of psychopathology; and other cognitive organic factors [6]. However, since each organ has a different treatment course before and after transplantation, the SIPAT results should be interpreted in an organ-specific manner. Therefore, we presented the basic data on SIPAT scores, by organ, for Japanese participants eligible for this study. Furthermore, although the psychosocial background is expected to be different between transplantations performed with organs from cadaveric and living donors, previous studies have not clarified the characteristics of the result profiles, such as which SIPAT sub-item domain scores are higher for each organ.

This exploratory research aimed to present the SIPAT scores of organ transplant recipient candidates in Japan and to examine the differences in the scores among different organs. Additionally, by comparing the SIPAT scores using organ-specific demographic data, we aimed to determine the characteristics of SIPAT scores for each organ. Thus, this study presents basic data that could be used to establish the relation between SIPAT scores and post-transplantation psychosocial outcomes as well as organ-specific outcomes.

## Methods

### Subjects

Candidates for organ transplant at the Tokyo Women’s Medical University Hospital were eligible for participation. All candidates underwent a psychosocial assessment interview before transplant surgery. Consecutive heart (26), liver (72), and kidney transplant (83) recipients who underwent pre-transplant psychosocial assessment from September 2018 to December 2021 were eligible. Of the 181 candidates, consent to participate in the study was not obtained from nine, one did not speak Japanese, and four under 20 years of age were unable to obtain consent from a surrogate; thus, they were excluded. There were no candidates judged ineligible for transplantation due to poor psychosocial factors during study period. However, we cannot rule out the possibility that some cases were deemed ineligible by the transplant team before they were referred to our pre-transplant interviews.

### The Japanese version of SIPAT

The SIPAT has demonstrated excellent inter-rater reliability (Pearson’s correlation coefficient = 0.85) and predictive ability for outcomes in previous studies [14]. The reliability and validity of the SIPAT-J have been established previously [6]. The SIPAT-J assesses 18 items, classified into four domains: (A) patient’s readiness and illness management level, (B) social support system level of readiness, (C) psychological stability and psychopathology, and (D) lifestyle and effect of substance use. Table 1 shows psychosocial domains and factors measured by the SIPAT. In previous studies, patients were classified into the following groups according to their total scores: excellent (0–6), good (7–20), minimally acceptable (21–39), poor (40–69), or high-risk (≥ 70). The SIPAT also includes a list of contraindications [14, 16].

**Table 1:** Psychosocial Domains and Factors Measured by the SIPAT

SIPAT A. Patient’s Readiness Level and Illness Management (5 items)	
	Item 1: Knowledge and understanding of medical illness process (that caused specific organ failure)
	Item 2: Knowledge and understanding of the process of transplantation
	Item 3: Willingness/desire for treatment (transplant)
	Item 4: History of treatment adherence/compliance (pertinent to medical issues)
	Item 5: Lifestyle factors (including diet, exercise, fluid restrictions, and habits, according to organ system)
SIPAT B. Social Support System Level of Readiness (3 items)	
	Item 6: Availability of social support system
	Item 7: Functionality of social support system
	Item 8: Appropriateness of physical living space and environment
SIPAT C. Psychological Stability and Psychopathology (5 items)	
	Item 9: Presence of psychopathology (other than personality disorders and organic psychopathology)
	Item 10: History of organic psychopathology or neurocognitive impairment (i.e., illness or medication induced psychopathology)
	Item 11: Influence of personality traits versus disorder
	Item 12: Effect of truthfulness versus deceptive behavior
	Item 13: Overall risk for psychopathology
SIPAT D. Lifestyle and Effect of Substance Use (5 items)	
	Item 14: Alcohol use, abuse, and dependence
	Item 15: Alcohol abuse—risk for recidivism
	Item 16: Illicit substance abuse and dependence
	Item 17: Illicit substance abuse—risk for recidivism
	Item 18: Nicotine use, abuse, and dependence

### Procedures

For all organs, candidates were deemed eligible for transplantation, informed consent was obtained, a psychiatric interview was requested, and the SIPAT was conducted. The evaluators, including one psychiatrist and three clinical psychologists, involved in the transplant care of the patients, independently and blindly applied SIPAT-J to the medical records of anonymized transplant recipient candidates. The evaluators were provided anonymized records, including the results of interviews with psychiatrists, clinical psychologists, and transplant coordinators.

### Statistical analyses

The level of significance for the statistical analysis was set at P < .05 (two-sided). Differences in total SIPAT scores, SIPAT sub-scores, and SIPAT scores for specific organs were compared using the Kruskal–Wallis test. Multiple comparisons were made between groups on measures for which significant differences were found using the Dunn–Bonferroni method. Kruskal–Wallis and Mann–Whitney U tests were used for comparisons of SIPAT scores by demographic data. Nonparametric tests were used in this study. The calculation of the sample size was based on the calculations in the ANOVA. The total sample size was 159, calculated with a number of groups of 3, significance level of 0.05, power of 80%, and effect size of 0.25. Statistical analyses were performed using the IBM SPSS Statistics version 28 (IBM Corp., Armonk, NY, USA).

## Results

### Participant characteristics

The sample consisted of 167 transplant recipient candidates (25 heart, 71 liver, and 71 kidney). The demographic information of the eligible participants is shown in Table 2. The mean age of the participants was 48.87 years. Of the eligible participants, 36.53% were female, 55.09% had education below high school level, 58.34% were employed, 69.46% were married, and 16.77% had a history of psychiatric treatment. The rate of living donor transplantation was 62.28% for all organs, 0% for the heart, 46.48% for the liver, and 100% for the kidney.

**Table 2:** Participant Demographics

	All (n = 167)	Heart (n = 25)	Liver (n = 71)	Kidney (n = 71)
Age, mean (SD), years	48.87 (12.36)	43.24 (13.27)	51.25 (10.27)	48.48 (13.39)
Female (%)	61(36.53)	6 (24.00)	35 (49.30)	20 (28.17)
Highest level of education obtained				
≤High school (%)	92 (55.09)	13 (52.00)	47 (66.20)	32 (45.07)
>High school (%)	75 (44.91)	12 (48.00)	24 (33.80)	39 (54.93)
Psychiatric treatment (%)	28 (16.77)	6 (24.00)	15 (21.13)	7 (9.86)
Employed (%)	115 (58.34)	17 (68.00)	41 (57.75)	57 (80.28)
Marital status				
Married or in a stable relationship (%)	116 (69.46)	17 (68.00)	48 (67.61)	51 (71.83)
Common-law marriage (%)	3 (1.80)	N.A.	2 (2.82)	1 (1.41)
Single (%)	37 (22.16)	8 (32.00)	14 (19.72)	15 (21.13)
Divorced (%)	10 (5.99)	N.A.	6 (8.45)	4 (5.63)
Widowed (%)	1 (0.6)	N.A.	1 (1.41)	N.A.
Living donor (%)	104 (62.28)	N.A.	33(46.48)	71 (100.00)

N.A. = not applicable; SD = standard deviation

### SIPAT scores by demographic characteristics

Table 3 shows SIPAT scores based on the demographic data. For all organs as well as each organ, there were no differences in SIPAT scores according to age, sex, and educational history. Contrastingly, for all organs, candidates with a history of psychiatric treatment had significantly higher SIPAT scores than those without a history of psychiatric treatment. Those who were not employed also had significantly higher SIPAT scores than those who were employed.

**Table 3:** SIPAT scores by demographic characteristics

	All (n = 167)				Heart (n = 25)				Liver (n = 71)				Kidney (n = 71)		
Demographic Characteristics	N (%)	SIPAT Total Mean(SD)	*P* Value		N (%)	SIPAT Total Mean(SD)	*P* Value		N (%)	SIPAT Total Mean(SD)	*P* Value		N (%)	SIPAT Total Mean(SD)	*P* Value
Age years															
< 30	14 (8.4)	18.14 (7.10)	.641^a^		6(24.0)	18.00 (5.06)	.832^a^		3 (4.2)	22.33 (11.24)	.616^a^		5 (7.0)	15.80(6.98)	.554^a^
30–59	124 (74.3)	20.43 (8.00)			17 (68.0)	16.76 (6.14)			54 (76.1)	22.76 (9.25)			53 (74.6)	19.23(6.41)	
> 60	29 (17.4)	19.24 (5.86)			2 (8.0)	17.00 (9.90)			14 (19.7)	19.57 (6.31)			13 (18.3)	19.23(5.28)	
Sex															
Female	61 (36.5)	19.02 (7.08)	.281^b^		6 (24.0)	15.33 (7.98)	.333^b^		35 (49.3)	19.91 (7.37)	.062^b^		20 (28.2)	18.55(6.49)	.725^b^
Male	106 (63.5)	20.61 (7.85)			19 (76.0)	17.63 (5.62)			36 (50.7)	24.25 (9.62)			51 (71.8)	19.16(6.19)	
Highest level of education obtained															
≤High school	92 (55.1)	20.48 (7.07)	.144^b^		13 (52.0)	17.38 (7.06)	.894^b^		47 (66.2)	21.91 (7.12)	.507^b^		32 (45.1)	19.63(6.66)	.418^b^
>High school	75 (44.9)	19.48 (8.21)			12 (48.0)	16.75 (4.62)			24 (33.8)	22.50 (11.58)			39 (54.9)	18.46(5.90)	
Psychiatric treatment															
Yes	28 (16.8)	26.46 (10.12)	< .001^b^		6 (24.0)	20.00 (8.74)	.555^b^		15 (21.1)	29.13 (11.64)	.006^b^		7 (9.9)	26.29(4.68)	.001^b^
No	139 (83.2)	18.73 (6.27)			19 (76.0)	16.16 (4.63)			56 (78.9)	20.23 (6.86)			64 (90.1)	18.19(5.88)	
Employed															
Yes	115 (68.9)	18.93 (6.41)	.016^b^		17 (68.0)	17.65 (5.85)	.669^b^		41(57.7)	20.10 (7.24)	.034^b^		57 (80.3)	18.47(5.90)	.141^b^
No	52 (31.1)	22.46 (9.34)			8 (32.0)	15.88 (6.22)			30 (42.3)	24.87 (10.06)			14 (19.7)	21.07(7.31)	

SD: standard deviation. SIPAT: Stanford Integrated Psychosocial Assessment for Transplantation. P: *P* Value was based on either Kruskal–Wallis test ^a^ or Mann–Whitney U test ^b^

### Comparison of SIPAT scores by organ

**Fig. 1:**
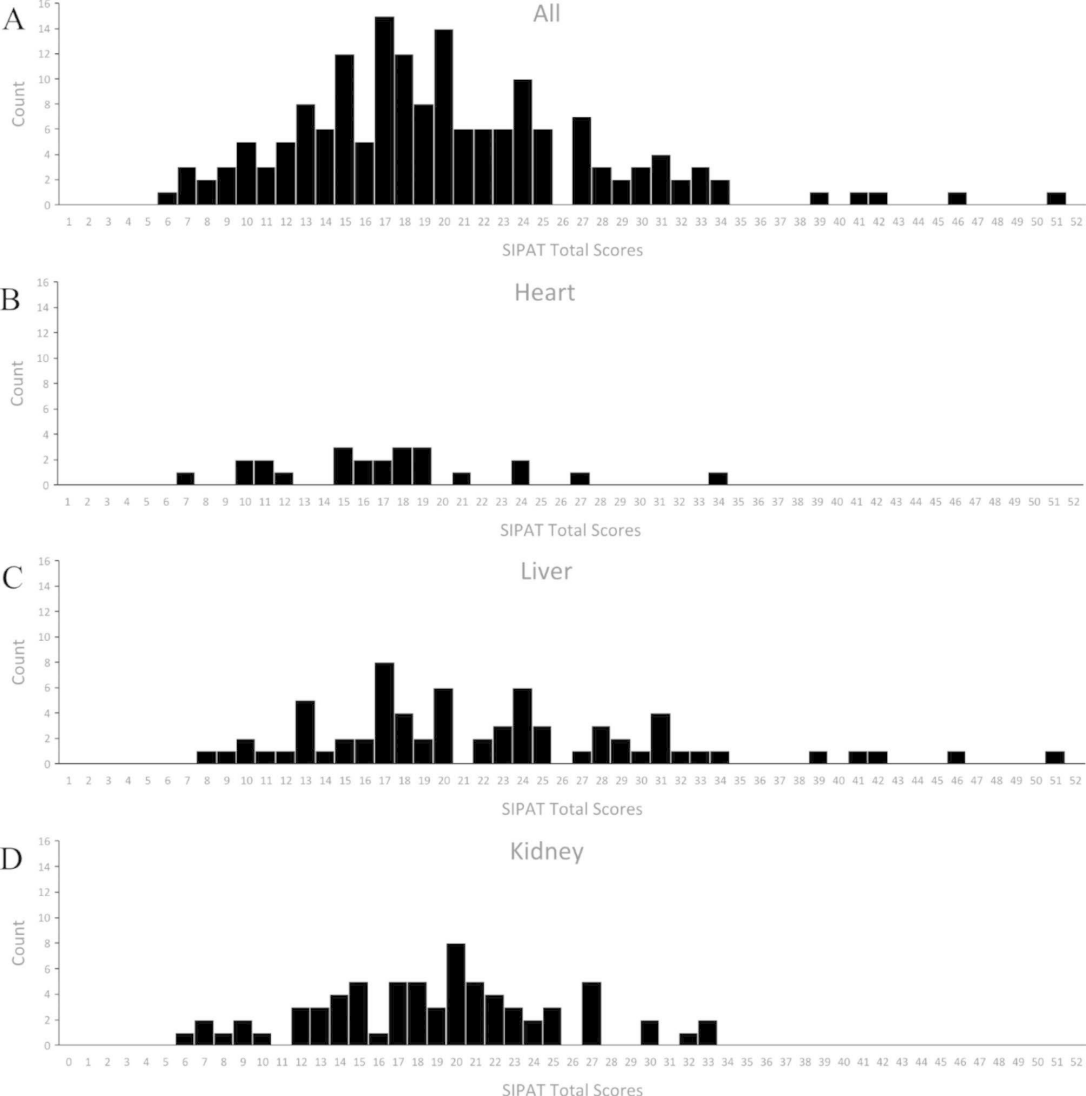
Distribution of the total Stanford Integrated Psychosocial Assessment for Transplantation scores **A**: (All) Distribution of the total Stanford Integrated Psychosocial Assessment for Transplantation scores for all organs. **B**: (Heart) Distribution of the total Stanford Integrated Psychosocial Assessment for Transplantation scores for heart. **C**: (Liver) Distribution of the total Stanford Integrated Psychosocial Assessment for Transplantation scores for liver. **D**: (Kidney) Distribution of the total Stanford Integrated Psychosocial Assessment for Transplantation scores for kidney

Table 4 shows the results of the SIPAT total and subscale scores for each transplant organ. The mean SIPAT total score was 20.03. The mean score for the liver recipient candidates was higher than that for the heart recipient candidates. The SIPAT B (social support system) score was significantly higher for the liver and kidney recipient candidates than for the heart recipient candidates, and the SIPAT C (psychological stability and psychopathology) score was significantly higher for liver recipient candidates than for kidney recipient candidates. The SIPAT A (patient readiness level) and SIPAT D (lifestyle and effect of substance use) scores were not significantly different among the organs. Figure 1 presents the distribution of the total SIPAT scores and organ-specific SIPAT scores.

**Table 4:** Summary of total and subscale SIPAT scores

	All (SD) (n = 167)	Heart (SD) (n = 25)	Liver (SD) (n = 71)	Kidney (SD) (n = 71)	*P* Value	Multiple comparison
SIPAT Total	20.03 (7.60)	17.08 (5.90)	22.11 (8.80)	18.99 (6.24)	0.019	Liver > Heart
SIPAT A (Patient’s readiness and illness management level)	7.04 (3.20)	6.08 (2.45)	7.15 (3.42)	7.25 (3.18)	0.378	
SIPAT B (Social support system level of readiness)	6.37 (3.03)	4.84 (2.25)	6.70 (3.04)	6.58 (3.12)	0.021	Liver = Kidney >Heart
SIPAT C (Psychological stability and psychopathology)	2.80 (3.08)	2.40 (2.29)	3.85 (3.74)	1.90 (2.19)	0.002	Liver > Kidney
SIPAT D (Lifestyle and effect of substance use)	3.82 (2.76)	3.76 (2.24)	4.41 (3.42)	3.25 (2.01)	0.313	

SD: standard deviation. SIPAT: Stanford Integrated Psychosocial Assessment for Transplantation

Table 5 shows the scores for the question items. For question 3 (willingness/desire for treatment [transplant]), the liver and kidney recipient candidates had higher scores than the heart recipient candidates. The scores for question 4 (history of treatment adherence/compliance [pertinent to medical issues]) were significantly higher for the kidney than for heart recipient candidates. The scores for question 6 (availability of social support system) were higher for the liver than for cardiac recipient candidates. The scores for question 8 (appropriateness of physical living space and environment) were higher for the liver and kidney recipient candidates than for cardiac recipient candidates. The scores for question 10 (history of organic psychopathology or neurocognitive impairment, i.e., illness or medication-induced psychopathology) were higher for the liver and kidney than for heart recipient candidates. The scores for question 13 (overall risk for psychopathology) were higher for the liver than for heart recipient candidates.

**Table 5:** Summary of SIPAT question items

Question items	All (SD) (n = 167)	Heart (SD) (n = 25)	Liver (SD) (n = 71)	Kidney (SD) (n = 71)	*P* Value	Multiple comparison
Item 1	1.08 (0.65)	0.84 (0.47)	1.17 (0.76)	1.07 (0.57)	0.125	
Item 2	1.33 (0.71)	1.28 (0.61)	1.46 (0.86)	1.21 (0.53)	0.161	
Item 3	0.78 (0.79)	1.28 (0.84)	0.80 (0.77)	0.59 (0.71)	< 0.001	Heart > Liver, Heart > Kidney
Item 4	2.60 (1.84)	1.60 (1.29)	2.54 (1.79)	3.01 (1.94)	0.005	Kidney > Heart
Item 5	1.25 (0.99)	1.08 (0.76)	1.18 (1.03)	1.37 (1.00)	0.273	
Item 6	2.49 (1.80)	1.52 (1.33)	2.39 (1.81)	2.93 (1.82)	0.003	Kidney > Heart
Item 7	2.43 (1.48)	2.16 (1.28)	2.65 (1.50)	2.31 (1.50)	0.332	
Item 8	1.45 (0.66)	1.16 (0.62)	1.66 (0.58)	1.34 (0.68)	< 0.001	Liver > Heart, Liver > Kidney
Item 9	0.40 (0.94)	0.48 (0.87)	0.54 (1.12)	0.23 (0.72)	0.117	
Item 9 A	0.33 (0.75)	0.36 (0.64)	0.41 (0.86)	0.24 (0.67)	0.278	
Item 9B	0.22 (0.49)	0.24 (0.44)	0.18 (0.49)	0.24 (0.52)	0.541	
Item 10	0.31 (0.86)	0.00 (0.00)	0.62 (1.19)	0.10 (0.42)	< 0.001	Liver > Heart, Kidney > Heart
Item 10 A	0.68 (0.76)	0.64 (0.70)	0.93 (0.82)	0.45 (0.65)	0.001	Liver > Heart, Kidney > Heart
Item 11	0.03 (0.67)	0.00 (0.00)	0.07 (0.31)	0.00 (0.00)	0.064	
Item 12	0.08 (0.67)	0.00 (0.00)	0.17 (1.00)	0.03 (0.24)	0.622	
Item 13	0.76 (0.65)	0.68 (0.56)	0.93 (0.68)	0.62 (0.62)	0.019	Liver > Heart
Item 14	2.00 (1.42)	1.92 (1.22)	2.34 (1.72)	1.69 (1.05)	0.082	
Item 15	0.99 (0.70)	0.92 (0.49)	1.17 (0.85)	0.85 (0.55)	0.063	
Item 16	0.02 (0.22)	0.00 (0.00)	0.06 (0.33)	0.00 (0.00)	0.257	
Item 17	0.02 (0.19)	0.00 (0.00)	0.04 (0.26)	0.01 (0.12)	0.625	
Item 18	0.78 (1.04)	0.92 (1.04)	0.80 (1.06)	0.70 (1.02)	0.587	

SD: standard deviation. SIPAT: Stanford Integrated Psychosocial Assessment for Transplantation

## Discussion

The purpose of this study was to present basic data that can be used to establish the relation between SIPAT scores and post-transplantation psychosocial outcomes as well as organ-specific outcomes. To achieve this, we determined the distribution of SIPAT scores among organ transplant recipient candidates, examined the differences in scores by organ, and compared the SIPAT scores for each organ according to demographic data. We found that the liver recipient candidates had higher scores than heart recipient candidates. Regarding subscale scores, the liver and kidney recipient candidates had higher scores than heart recipient candidates on SIPAT B (social support system). The liver recipient candidates had higher scores than kidney recipient candidates on SIPAT C (psychological stability and psychopathology). Additionally, recipient candidates with a history of psychiatric treatment and those who were not employed had very poor overall SIPAT scores. However, there were no differences in SIPAT scores by age, sex, or educational background.

### Comparison of SIPAT total scores by organ

Previous studies on SIPAT presented data from several countries including the United States, Spain, Italy, and Thailand. However, many previous studies described only the total SIPAT scores or score distributions [16, 17, 21, 26] and few studies provided comparisons of SIPAT scores among different organs. The total SIPAT score in the present study was 20.03. The total SIPAT score in a previous Thai study of heart, liver, and kidney transplant recipient candidates was 19.65 [27], and the total score in a previous Spanish study of heart, liver, and allogeneic hematopoietic stem cell transplant recipient candidates was 26.0 [21]. The total SIPAT score in a previous American study of heart, lung, liver, and kidney recipient candidates was 12.9 [16]. Thus, differences in total SIPAT scores have been observed in previous studies. It is unclear whether the differences were because of the characteristics of the organs, transplantation conditions in the countries, evaluator, or translation from English to other languages. For these reasons, we believe that for the SIPAT to be used in clinical practice, it is necessary to indicate the evaluation criteria for each country. In our study, the liver recipient candidates had significantly higher SIPAT scores than the heart recipient candidates. In a Thai SIPAT study on the same organs as those in the present study [27], the heart and liver recipient candidates had higher scores than kidney recipient candidates. The reasons for the differences in SIPAT scores by liver candidates were higher scores on SIPAT question items 8,10 and 13, which may reflect the history of alcohol abuse and hepatic encephalopathy or poor living space and environment. Therefore, information and education from transplant teams to recipient candidates should be provided based on the organ-specific trait of higher SIPAT scores. Furthermore, it is necessary to equalize the support provided by transplant teams to the recipient candidates for different organs. Because liver recipients showed higher SIPAT scores compared to heart and kidney recipients in Japan, liver recipients require more support based on SIPAT.

A comparison of SIPAT scores based on demographic data showed that scores of recipient candidates with a history of psychiatric treatment for all organs were higher than the scores of recipient candidates with no history of psychiatric treatment. In a study of kidney transplant recipients in the United States, men had higher scores than women, those with renal impairment secondary to hypertension had higher scores than those with renal impairment because of other causes, and those with low education levels had higher scores than those with high school education [15]. In this study, however, there were no differences in scores based on sex and educational background.

### Comparison of SIPAT subscale scores by organ

The SIPAT subscales are classified into four domains [14]. As with the total scores, differences in subscale scores between the organs are expected but have rarely been noted in previous studies. The analysis of the subscales revealed that the liver and kidney recipient candidates scored significantly higher than heart recipient candidates on SIPAT B, and the liver recipient candidates scored significantly higher than the kidney recipient candidates on SIPAT C. These organ-specific differences may be explained by the question items. The liver recipient candidates scored higher on items of residential settings (8), organic psychiatric disorders (10), cognitive assessment (11), and overall risk of psychiatric problems (13). In a previous study in Thailand, the scores on SIPAT A were higher for heart and liver recipient candidates than for kidney recipients, the scores on SIPAT B were higher for heart recipient candidates than for liver and kidney recipient candidates, and the scores on SIPAT D were higher for liver recipient candidates [27]. The difference between Japanese and Thai results in the scores of heart recipient candidates may reflect the differences in transplant-related education provided to recipient candidates in the two countries and differences in the severity of the condition of the eligible patients. Another peculiarity of the Japanese transplant situation is the long waiting period for brain-dead donors [28]. The SIPAT scores of the heart recipient candidates in this study were low for the items of medical visits and adherence (4) and availability of social support systems (6). Heart transplantation candidates need to undergo particularly rigorous psychosocial evaluations including good adherence to medical visits and social support [30]. These issues are due to few heart transplant donors in Japan. The transplant candidates in this study were patients who were judged by the transplant team to be likely candidates for organ transplantation. Therefore, it is possible that patients were not placed on the transplant waiting list because the transplant team determined that the patient was not suitable for organ transplantation because of lack of social support or non-adherence issues. It is possible that patients with high psychosocial risk (i.e., high scores on the SIPAT) were not included in this study.

### Limitations and future steps/research

This study has several limitations. First, this was a single-center study. Although the facility performs a large number of organ transplantations, there are many other transplantation facilities in Japan. There may be differences in patient education among the facilities, and these differences may affect the SIPAT scores. Furthermore, our analysis did not consider differences by facility, including regional differences. Second, our analysis only included heart, liver, and kidney transplant recipient candidates; therefore, the results may not be applicable to other organs. Considering the application of SIPAT in recipient candidates for a wide range of organs, it is necessary to clarify the characteristics of SIPAT for organs that were not included in this study. In addition, many heart recipient candidates in Japan are required to wait for long periods, during which time they may undergo implantation of ventricular assist devices [31, 32]. Therefore, we believe it is necessary to clarify the SIPAT scores specific to patients with ventricular assist devices on the transplant waiting list. Third, although this study discussed differences in SIPAT scores by organ, it is possible that in clinical practice, there may be differences in SIPAT scores between the time of the interview and proximate time before transplant surgery when more detailed information is given. Therefore, SIPAT may need to be re-administered in order to assess the psychosocial status of a transplant candidate in a timely manner.

Our study presented the SIPAT scores of organ transplant recipient candidates in Japan and evaluated the differences in the scores by each organ. Once it is clear that SIPAT-J predicts post-transplant outcomes, the early evaluation of recipient candidates followed by timely interventions can improve post-transplant outcomes. Larger datasets are needed to clarify the association between SIPAT and post-transplant outcomes. Finally, although previous studies have used a four-point scale based on the total SIPAT score, it is not clear whether this evaluation method is available in Japan. Comparisons with other countries showed different mean scores and distributions; therefore, the cut-off scores of SIPAT-J need to be confirmed for the Japanese population.

## Conclusions

This study is the first to present the SIPAT-J scores, which will provide important basic data for future SIPAT studies. The results showed differences in the SIPAT-J total and subscale scores among the transplant organs, indicating that specific psychosocial issues should be addressed before the transplantation of specific organs. Interventions such as information provision and patient education based on SIPAT assessment results for each organ may improve the post-transplant outcomes of recipients. Future prospective studies should clarify whether SIPAT assessment results predict physical and psychosocial outcomes by organ type. In addition, organ-specific cut-off values need to be established for the Japanese population.

## Data Availability

The data and codes that support the findings of this study are available on request from the corresponding author. The data are not publicly available owing to privacy or ethical restrictions.

## Abbreviations

N.A.: Not applicable
PACT: The Psychosocial Assessment of Candidates for Transplant
SIPAT: The Stanford Integrated Psychosocial Assessment for Transplantation
SD: Standard deviation
